# Hydroxychloroquine induces apoptosis of myeloid-derived suppressor cells via up-regulation of CD81 contributing to alleviate lupus symptoms

**DOI:** 10.1186/s10020-022-00493-6

**Published:** 2022-06-15

**Authors:** Jiali Ni, Haiyan Zhu, Li Lu, Zihe Zhao, Jiaxuan Jiang, Xiaokang You, Yuzhu Wang, Yuliang Ma, Zirui Yang, Yayi Hou, Huan Dou

**Affiliations:** 1 The State Key Laboratory of Pharmaceutical Biotechnology, Division of Immunology, Medical School, Nanjing University, Nanjing, 210093 People’s Republic of China; 2 Jiangsu Key Laboratory of Molecular Medicine, Nanjing, 210093 People’s Republic of China

**Keywords:** Hydroxychloroquine, SLE, IMQ-induced lupus, MDSCs, Apoptosis, CD81

## Abstract

**Background:**

Systemic lupus erythematosus (SLE) is a chronic autoimmune disorder that results from widespread immune complex deposition and secondary tissue injury. Hydroxychloroquine (HCQ) has been used clinically to treat SLE, while its exact mechanism has still remained elusive. Some studies have shown that myeloid-derived suppressor cells (MDSCs) play a vital role in the regulation of SLE. In this study, we aimed to explore the effects of HCQ on the apoptosis of MDSCs in lupus mice and its possible molecular regulatory mechanism.

**Methods:**

We constructed the imiquimod (IMQ)-induced lupus model in mice. The proportion and apoptosis of MDSCs were measured by flow cytometry. CD81-overexpressed adeno-associated virus was intraperitoneally injected into the lupus mice. We also transfected the CD81 siRNA into bone marrow-derived MDSCs, and employed qRT-PCR and Western blotting to quantify the level of CD81.

**Results:**

The results showed that HCQ ameliorated IMQ-induced lupus symptoms, and simultaneously inhibited the expansion of MDSCs. In particular, HCQ induced the apoptosis of MDSCs, and also up-regulated the expression level of CD81 in MDSCs, which might indicate the relationship between the expression level of CD81 and the apoptosis of MDSCs. CD81 was further confirmed to participate in the apoptosis of MDSCs and lupus disease progression by overexpressing CD81 in vivo. Molecular docking experiment further proved the targeting effect of HCQ on CD81. And then we interfered CD81 in bone marrow derived MDSCs in vitro, and it was revealed that HCQ rescued the decreased expression level of CD81 and relieved the immune imbalance of Th17/Treg cells.

**Conclusion:**

In summary, HCQ promoted the apoptosis of MDSCs by up-regulating the expression level of CD81 in MDSCs, and ultimately alleviated lupus symptoms. Our results may assist scholars to develop further effective therapies for SLE.

**Supplementary Information:**

The online version contains supplementary material available at 10.1186/s10020-022-00493-6.

## Introduction

Hydroxychloroquine (HCQ), belongs to the 4­aminoquinolines class with antimalarial activity and an immunomodulatory effect (Schrezenmeier and Dörner 2020; Munguía-Realpozo et al., 2021; Gupta et al. 2022). It has also been used clinically in the treatment of systemic lupus erythematosus (SLE) (Costedoat-Chalumeau et al. 2014; Broder et al. 2021), downregulating pro-inflammatory cytokines and immunoglobulin, eliminating autoreactive lymphocytes, ultimately improves patient survival and relieves severe lupus symptoms (Takamasu et al. 2019; Ponticelli and Moroni 2017; Willis et al. 2012; Rainsford et al. 2015). Some studies have shown that HCQ mainly accumulates in acidic vesicular lysosomes, affects the functions of proteasomes by increasing intracellular vacuolar pH, and also inhibits the functions of endolysosomes regulating autophagy, calcium signaling, and toll-like receptor (TLR)-mediated immune responses (Wu et al. 2017; Wallace et al. 2012; Nirk et al. 2020). However, the mechanism of HCQ in treating SLE has not been fully clarified, and the possible therapeutic target remains unclear.

Myeloid-derived suppressor cells (MDSCs) are a heterogeneous population of immature myeloid cells originated from myeloid progenitor cells, and can be classified into two subsets, granulocyte-like MDSCs (G-MDSCs) and monocyte-like MDCSs (M-MDSCs) (Lees et al. 2011). Compared to other myeloid cells, MDSCs exhibited an immunosuppressive capacity to inhibit proliferation of T cells and alter regulatory T cell (Treg) by generating effector molecules, such as reactive oxygen species (ROS), inducible nitric oxide synthase (iNOS), interleukin-6 (IL-6), and transforming growth factor-β (TGF-β) (Gabrilovich et al. 2017; Vlachou et al. 2016). Expansion of MDSCs is generally associated with inflammatory processes that emerge in response to stable immunological stress, altering both magnitude and quality of the myelopoietic output (Veglia et al. 2018; Gabrilovich and Nagaraj 2009). The significant accumulation of MDSCs has been found to be positively correlated to disease activity in SLE patients. Our laboratory and other researchers reported the same symptom in various mouse models of lupus (Dong et al. 2015; Wu et al. 2016; Li et al. 2019a, b; Ji et al. 2016; Li et al. 2019a, b). In previous studies, the of MDSCs in male (NZB × NZW) F1 mice constitutively increased compared with that in female mice and that was regulated by testosterone (Trigunaite et al. 2013). Various effects of MDSCs on target cells have proven to be a critical pathogenic factor in SLE, including dysregulation of T helper type 17 (Th17) cells and differentiation of Treg (Ji et al. 2016). MDSCs can also induce podocyte injury by increasing the levels of ROS in lupus nephritis (Zhang et al. 2018a, b). In conclusion, investigating the regulation of MDSCs may provide new insights into the therapy of SLE.

CD81 is a member of the tetraspanin family, known as a receptor of hepatitis C virus (HCV), which can mediate HCV envelope protein 2 (E2) to bind to Raji cells (Ströh et al. 2018; Chen et al. 2011). Transfection of CD81 into melanoma cells lacking endogenous CD81 expression could significantly enhance the migrating, invasion, and metastatic abilities of melanoma cells (Hong et al. 2014; Bari et al. 2011). CD81 in trophoblasts can induce the imbalance between Th17/Treg cells by promoting the expression of IL-6 in preeclampsia and aggravating the disease progression (Ding et al. 2019). And restoration of CD81 expression induced a G1 cycle arrest and apoptosis in gastric tumor (Yoo et al. 2013). Furthermore, the expression of CD81 in B cells from SLE patients was down-regulated, and that could be correlated with abnormal activation of B cells (especially antibody secreting cells) and disease activity (especially at the active stage) (Henriques et al. 2016; Abu-Zahab et al. 2017; Dang et al. 2022). It suggested that CD81 could be a potential indicator for the diagnosis of SLE.

Our study aimed to assess the effect of HCQ on MDSCs during the treatment of SLE. HCQ inhibited the expansion of MDSCs, accompanied with the induced apoptosis and altered immunosuppressive function of MDSCs in imiquimod (IMQ)-induced lupus mice. Further research revealed that the role of CD81 participate in the apoptosis of MDSCs increased by HCQ. SLE-like symptoms were attenuated by up-regulating the expression level of CD81, and CD81 also increased the apoptosis of MDSCs directly. Hence, these findings demonstrated that HCQ could ameliorate lupus by inducing the apoptosis of MDSCs through upregulating the expression level of CD81, which further indicate the significance of MDSCs in lupus.

## Materials and methods

### Mice

Female C57BL/6 mice (age, 6–8 weeks old) were obtained from Changzhou Cavens Experimental Animal Co., Ltd. (Changzhou, China) and were maintained under specific pathogen-free (SPF) conditions at a light‐dark cycle (12:12 h light–dark cycle). Mice were acclimatized in housing conditions for at least 1 week. Next, mice were randomly divided into different groups (n = 6 per group). The skin on the back of right ear was treated with 1.25 mg 5% imiquimod cream (three times/week) to establish a mouse model of lupus (Yokogawa et al. 2014). During the next 10 weeks, mice received HCQ (60 mg/kg) by gavage (six times/week). Mice in the IMQ + Vector and IMQ + AAV-CD81 groups were given IMQ for establishing the mouse model of lupus, and CD81 overexpression adeno-associated virus (GeneChem, Shanghai, China) by intraperitoneal injection of 1 × 10^11^Ug virus/mice at the beginning of the model. All mice were eventually euthanized by asphyxiation via carbon dioxide (Conlee et al. 2005).

### Calculation of spleen index

Spleen index was calculated according to the formula: spleen index (mg/g) = spleen weight (mg)/live weight before slaughter (g).

### Generation and isolation of MDSCs

Bone marrow (BM) cells were isolated from mice as previously described by flushing femurs and tibiae (Ji et al. 2016). Then, BM cells were centrifuged and resuspended in a Roswell Park Memorial Institute (RPMI)-1640 medium (Gibico, Grand Island, NY, USA) supplemented with 10% fetal bovine serum (FBS; Gibico), murine IL-6, and granulocyte–macrophage colony-stimulating factor (GM-CSF) (both 40 ng/mL; Miltenyi Biotec, Auburn, CA, USA) and were cultured for 4 days. Spleen-derived MDSCs were isolated by the MDSC Isolation Kit (catalog no., 130-094-538; Miltenyi Biotec) according to the manufacturer’s instructions.

### Flow cytometry

To detect mouse MDSCs, cells, which were directly isolated from spleen and blood, were pre-incubated with PE Vio770-conjugated-CD11b (catalog no. 130-113-236; Miltenyi Biotec) and Alexa Fluor 647-conjugated-Gr-1 (catalog no. 108418; BioLegend, San Diego, CA, USA) antibodies, and were then incubated for 30 min at 4 °C in the dark. To detect mouse Th17 cells, cells were pre-treated in the RPMI-1640 medium, containing phorbol-12-myristate-13-acetate (PMA), ionomycin (Ion), and brefeldin A (BFA) (Beyotime, Shanghai, China). Afterward, the cells were stained with FITC-conjugated-CD4 (catalog no. 11-0041-82; eBioscience, San Diego, CA, USA) and PE-conjugated-IL-17A (catalog no. 506903; BioLegend) antibodies. To detect mouse Treg cells, cells were pre-incubated with FITC-conjugated-CD4, APC-conjugated-CD25 (catalog no. 17-0251-81; eBioscience), and PE-conjugated-Foxp3 (catalog no. 12-5773-80A; eBioscience) antibodies. In addition, the cells were stained with PE Vio770-conjugated-CD11b, Alexa Fluor 647-conjugated-Gr-1, and PE-conjugated-CD81 (catalog no. 104906; Biolegend) antibodies for detection of the expression level of CD81 in MDSCs from spleen and blood of mice.

Levels of ROS in MDSCs from spleen and blood of mice were detected using the ROS Assay Kit (Beyotime). Briefly, cells were pre-incubated with PE Vio770-conjugated-CD11b and Alexa Fluor 647-conjugated-Gr-1 antibodies, and then, incubated at 37 °C in an RPMI-1640 medium in the presence of 2.5 µM 2,7-Dichlorodihydrofluorescein diacetate (DCFDA), and simultaneously cultured with 1 µg/mL lipopolysaccharide (LPS) (Sigma-Aldrich, St. Louis, MO, USA) for 20 min. After that, the cells were washed with 1 mL RPMI-1640 medium twice and analyzed by flow cytometry.

The apoptosis of MDSCs was assessed by flow cytometry using an Annexin V-FITC Apoptosis Detection Kit (catalog no. FMS-AP-001; Fcmacs, Nanjing, China). In vivo, cells, which were directly isolated from spleen and blood, were pre-incubated with PE Vio770-conjugated-CD11b and Alexa Fluor 647-conjugated-Gr-1 antibodies. Then, the cells were stained with Annexin V-FITC at room temperature for 15 min in the dark. After removing the unbound Annexin V-FITC by centrifugation, the cells were resuspended in 500 μL binding buffer. The cells were analyzed by a flow cytometer. In vitro, BM-MDSCs were treated with different stimulations. Then, the rate of apoptosis of MDSCs was detected using the Annexin V-FITC Apoptosis Kit in vivo.

### Carboxyfluorescein diacetate succinimidyl ester (CFSE)-based assay

The CFSE-based assay was performed as described previously (Ji et al. 2016). Normal splenocytes (2 × 10^5^ cells/well) were labelled with 5 μM CFSE according to the manufacturer’s instructions. CFSE-labelled splenocytes were then co-cultured with purified splenic MDSCs at ratios of 2:1 or 4:1 in the presence of 4 μg/mL anti-CD3 monoclonal antibody and 2 μg/mL anti-CD28 monoclonal antibody in a 96-well flat-bottom plate for 72 h. On the 4th day, the proliferation of CD4^+^ T cells was measured by flow cytometry. These reagents were all purchased from eBioscience.

### Immunofluorescence staining

Frozen splenic sections of mice were stained with rat anti-mouse Ly-6G/Ly-6C(Gr-1) monoclonal antibody (eBioscience), followed by treatment with Alexa Fluor®647-conjugated donkey anti-rat IgG H&L antibody (Abcam, Cambridge, UK). The nucleus staining was performed with 4′,6-diamidino-2-phenylindole (DAPI) (Fcmacs). Finally, the coverslips were observed and captured by a laser scanning confocal microscope (FV3000; Olympus Corp., Tokyo, Japan).

The terminal deoxynucleotidyl transferase dUTP nick end labeling (TUNEL) assay was conducted with the in-situ cell death detection kit that contained fluorescein (Roche Molecular Biochemicals, Indianapolis, IN, U.S.A), according to the manufacturer’s instructions.

### Western blot analysis

The proteins were extracted by standard techniques described previously (Li et al. 2017). The protein concentrations were determined using the BCA Assay Kit (Thermo Fisher Scientific, Waltham, MA, USA). Then, the total proteins were separated by sodium dodecyl sulfate–polyacrylamide gel electrophoresis (SDS-PAGE), and transferred onto polyvinylidene fluoride (PVDF) membranes. Membranes were blocked in 5% bovine serum albumin (BSA) (Biosharp, Hefei, Anhui, China) dissolved in TBST (Tris-buffered saline, 0.1% Tween 20) for 2 h at room temperature, and incubated with indicated primary antibody overnight at 4 °C. After that, the membranes were incubated with appropriate horseradish peroxidase (HRP)-linked secondary antibody for 2 h at room temperature. Protein bands were visualized by ECL Plus reagents (Thermo Fisher Scientific). The images were captured by a minichem™ chemiluminescence imaging system (Sagecreation, Beijing, China). The gray values were analyzed by LANE 1D Analysis software.

### RNA extraction and quantitative reverse transcription polymerase chain reaction (qRT-PCR)

Total RNA was extracted from tissues and cells using TRIzol Reagent (Invitrogen, Carlsbad, CA, USA). The qRT-PCR was conducted as previous described (Zhao et al. 2018). The relative gene expression values were determined using the 2^−ΔΔCt^ method, and glyceraldehyde 3-phosphate dehydrogenase (GAPDH) was considered as an internal control. Primer sequences used for qRT-PCR are presented in Additional file 1: Table S1.

### Enzyme-linked immunosorbent assay (ELISA)

Urine of mice was collected and the total urinary protein was determined using a Mouse Albumin ELISA Quantitation Set (Bethyl Laboratories) according to the manufacturer’s instructions. The urine was applied at a dilution of 1:1000. Sera anti-dsDNA was analyzed using a Mouse ds-DNA Kit (Westang, Shanghai, China) and the sera was applied at a dilution of 1:10. Sera anti-IgG and anti-IgM were analyzed using a Mouse IgG total ELISA Kit and Mouse IgM ELISA Kit (Fcmacs), respectively, according to the manufacturer’s instructions and the sera was applied at a dilution of 1:500,000.

### Histological analysis and evaluation

The kidney tissues were fixed with 4% paraformaldehyde in phosphate-buffered saline (PBS). Paraffin-embedded samples were sectioned at 3 μm, and stained with hematoxylin and eosin (H&E) and periodic acid-Schiff (PAS) methods. Renal histopathological changes were quantitated with the method as described before (Fu et al. 2021). Pathological changes in the kidney comprised glomerular activity (i.e., glomerular proliferation, cellular crescents, hyaline deposits, and inflammatory cells) and tubulointerstitial activity (i.e., interstitial inflammation, and tubular distension). Sections were scored using a 0–3 scale for glomerular activity, as follows: 0: no lesions, 1: lesions in > 25% ofglomeruli, 2: lesions in 25–50% of glomeruli, and 3: lesions in > 50% of glomeruli. Tubulointerstitial activity was scored using a 0–4 scale, as follows: 0: no lesions, 1: lesions in 1–10%, 2: 11–25%, 3: > 25–50%, and 4: > 50–100%. The scores for individual pathological features from multiple mice were summed.

### Small interfering RNA (siRNA) cell transfections

Purified siRNA duplexes targeting the mouse CD81 gene were purchased from RiboBio Co., Ltd. (Guangzhou, China). To transfect BM-MDSCs, the medium was antibiotic-free RPMI-1640 supplemented with 10% FBS. Next, BM-MDSCs were transfected with RNA duplexes, by RFect^PM^ small nucleic acid transfection reagent (Baidai, Changzhou, China) according to the manufacturer’s instructions. After 72 h, cells were collected to treat with or without different concentrations of HCQ (2, 6, and 20 μg/mL). After 24 h, BM-MDSCs were harvested for detection.

### Differentiation of Th17 and Treg cells

Normal splenocytes were stimulated by anti-CD3 (5 μg/mL) and anti-CD28 (5 μg/mL) in the culture with hTGF-β (2.5 ng/mL) and IL-6 (20 ng/mL) in 24-well plates. In addition, pre-treated BM-MDSCs were added to the culture on day 0 at a ratio of 1:1 and cells were cultured in triplicate in culture medium. After 72 h, cells were analyzed by flow cytometry.

Normal splenocytes were cultured with anti-CD3 (5 μg/mL) and anti-CD28 (5 μg/mL) mAbs in the presence of TGF-β (5 ng/mL) and IL-2 (2 ng/mL) in a 24-well plate in a complete RPMI medium (5 × 10^5^ cells/well). Besides, pretreated BM-MDSCs were added to the culture on day 0 at a ratio of 1:1. After 72 h, we detected cells as mentioned in “Generation and isolation of MDSCs” section.

### Molecular docking

Molecular docking was conducted using Molecular Operating Environment (MOE) software (Ver. 2019). The three-dimensional (3D) structure of HCQ was downloaded from PubChem database. The 3D structure of the CD81 was downloaded from RCSB Protein Data Bank2. Prior to docking, the force field of AMBER10: EHT and the implicit solvation model of Reaction Field (R-field) were selected. MOE-Dock was used for molecular docking simulations of the HCQ with CD81. As the structure of CD81 is not complex, MOE’s Site Finder was utilized to calculate possible active sites in CD81 from the 3D atomic coordinates of 3X0F.

The docking workflow followed the “induced fit” protocol, in which the side chains of the receptor pocket were allowed to move according to ligand conformations, with a constraint on their positions. The weight used for tethering the side chain atoms to their original positions was 10. For each ligand, all docked poses were first ranked by the London dG scoring function, and then, a force field refinement was carried out on the top 20 poses, followed by a rescoring of GBVI/WSA dG. Molecular graphics were illustrated via MOE.

### Statistical analysis

The data were expressed as mean ± standard error of the mean (SEM) of three independent experiments, and each experiment was performed in triplicate. Differences between the two treatment groups were analyzed by the Student’s t-test, and comparisons among four groups were made by one-way analysis of variance (ANOVA). Multiple comparisons were assessed using post hoc Tukey test. A P-value lower than 0.05 (*P* < *0.05*) was considered statistically significant. All the statistical analyses were conducted using the GraphPad Prism 6.0 software (GraphPad Software Inc., San Diego, CA, USA). All experiments were performed at least thrice.

## Results

### HCQ inhibits the expansion of MDSCs in IMQ-induced lupus.

**Fig. 1:**
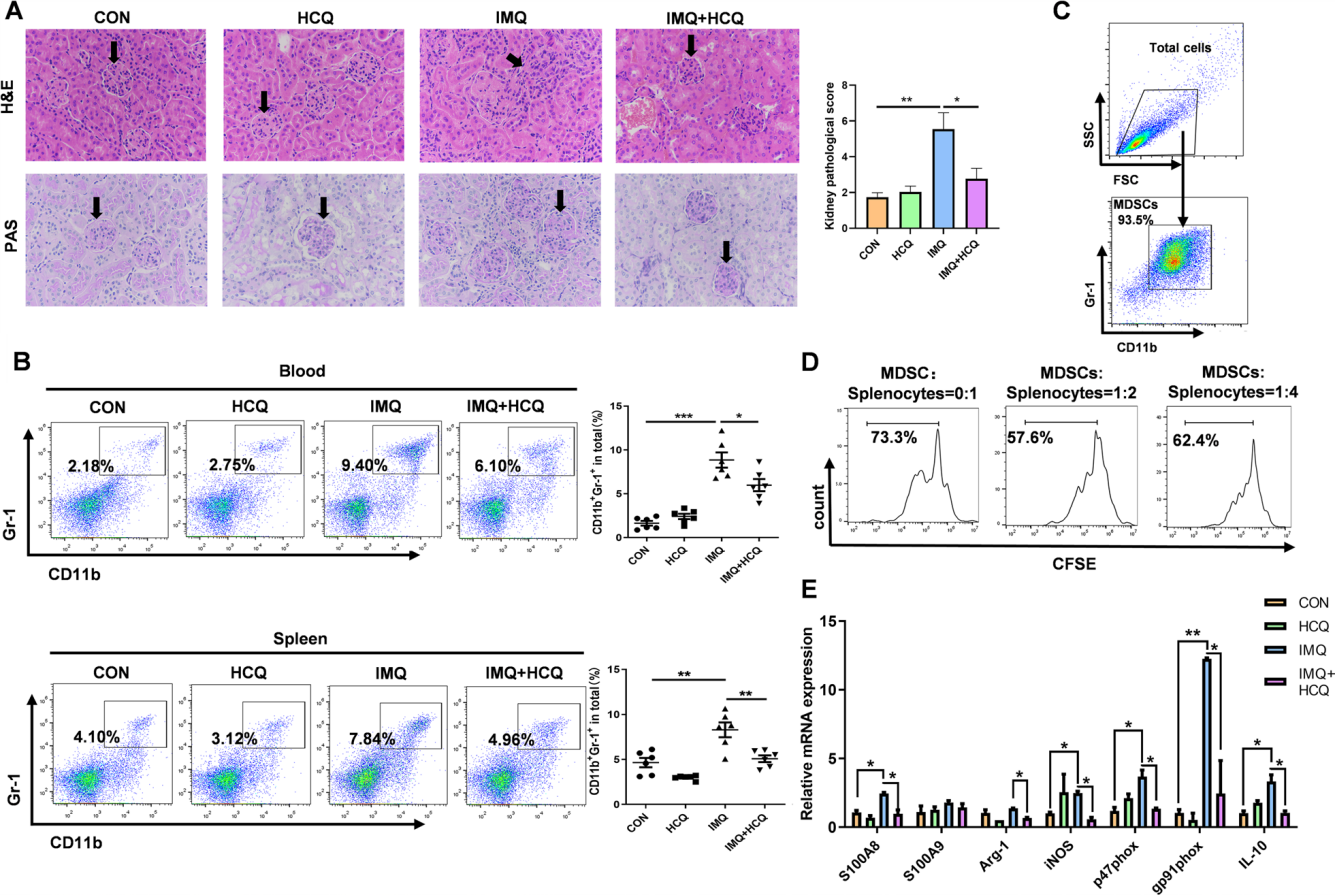
Amelioration of HCQ on IMQ-induced lupus inhibits MDSCs expansion. **A** Kidney histopathological evaluation by H&E and PAS staining in the control groups, IMQ groups, HCQ groups and IMQ + HCQ groups (n = 4 mice/group). Bar = 50 μm. **B** The frequency of MDSCs in blood and spleen and the statistical results of the frequency of MDSCs from control groups, IMQ groups, HCQ groups and IMQ + HCQ groups (n = 6 mice/group). **C** The purity of MDSCs isolated from spleen was assessed by a MDSC Isolation Kit. **D** Cells from spleen of normal C57BL/6J female mice were co‑cultured with or without MDSCs from mice of different groups in different ratios (MDSCs: Splenocytes) at 1:2 and 1:4, respectively for 3 days. CFSE was distributed equally in proliferative CD4^+^ T cells. **E** The mRNA expression levels of MDSCs function-related genes in MDSCs isolated from spleen was measured by qRT-PCR (n = 4 mice/group). Data represent the mean scores ± SEM. *P ≤ 0.05, **P ≤ 0.01, ***P ≤ 0.001

As HCQ has been clinically used to treat SLE, we verified its therapeutic effects on the murine IMQ-induced lupus model. HE and PAS staining displayed that glomerular proliferation, inflammatory cell infiltration and interstitial inflammation were relieved in the HCQ-treated lupus group (Fig. 1A). Splenomegaly was severe in IMQ-induced lupus mice and HCQ significantly relieved it (*P* < 0.05) (Additional file 1: Fig. S1A). As a prominent characteristic of lupus, the albumin level decreased with HCQ treatment (Additional file 1: Fig. S1B). In addition, high serum levels of antibodies have been reported to contribute to the pathogenesis of SLE. Notably, lupus mice treated with HCQ showed lower serum levels of IgG, IgM and anti-dsDNA (Additional file 1: Fig. S1C). Our previous studies have shown that the number of MDSCs was elevated in SLE patients, MRL/lpr mice, IMQ-induced and pristane-induced lupus mice (Li et al. 2019a, b; Ji et al. 2016; Li et al. 2019a, b). Then, the effects of HCQ on MDSCs were investigated by flow cytometry. The proportions of MDSCs in blood (*P* < 0.01) and spleen (*P* < 0.01) significantly decreased in the HCQ-treated lupus group (Fig. 1B).

To explore the effects of HCQ on the functions of MDSCs, we purified MDSCs from spleen in four groups of mice by using the MDSC Isolation Kit, and the sorting purity was nearly 94% (Fig. 1C). The proliferation of CD4^+^ T cells was inhibited by MDSCs in MRL/lpr mice (Iwata et al. 2010). MDSCs suppression assay showed that the proliferation capacity of CD4+ T cells was inhibited when splenic cells were co-cultured with sorted cells. It demonstrated the immunosuppressive function of CD11b^+^Gr-1^+^ cells (Fig. 1D). In addition to the inhibition of T cell proliferation, some gene were also involved in the regulation of MDSCs functions. The binding of cytosolic calcium-binding protein S100A8/S100A9 and gp91phox, p47phox led to the suppressive effects of MDSCs (Zhou et al. 2022). The subunits of NADPH oxidase 2 (Nox2) potentiates the activation of NADPH oxidase, which induced the production of reactive oxygen species (ROS) by MDSCs (Xue et al. 2019). The activation of T cells was also inhibited by the synthesis of arginase-1 (Arg-1) and inducible nitric oxide synthase (iNOS) (Raber et al. 2014). IL-10 produced by MDSCs has been shown to be involved in the development of Tregs and impair CD8^+^T cell function (Park et al. 2018). So, we detected these genes by qRT-PCR to trace the immunosuppressive function of MDSCs. HCQ decreased the mRNA expression of S100A8, Arg-1, iNOS, etc., and the inhibitory effects of HCQ on MDSCs function were evident (Fig. 1E). We also measured ROS levels of MDSCs in the blood and spleen, and HCQ-treated lupus group had the lower ROS levels of MDSCs (Additional file 1: Fig. S2A). Naïve CD4^+^ T cells are activated to differentiate into Th17 cells, which cause autoimmunity and inflammation, and Treg cells, which inhibit autoimmune response and maintain immune homeostasis (Lee 2018). Given the basis of our research, MDSCs could promote polarization of Th17 cells by secreting IL-1β, and induce production of ROS to inhibit differentiation of Treg cells (Ji et al. 2016). We further assessed the effects of HCQ on differentiation of Th17/Treg cells in lupus mice, and the immune imbalance of Th17/Treg cells in SLE was alleviated by HCQ treatment, which decreased the proportion of Th17 cells and increased the proportion of Treg cells (Additional file 1: Fig. S2B, C). These findings suggested that the ameliorative effects of HCQ on IMQ-induced lupus might be related to the suppression of MDSCs accumulation and function.

### HCQ induces apoptosis of MDSCs and increases the level of CD81 in MDSCs

**Fig. 2:**
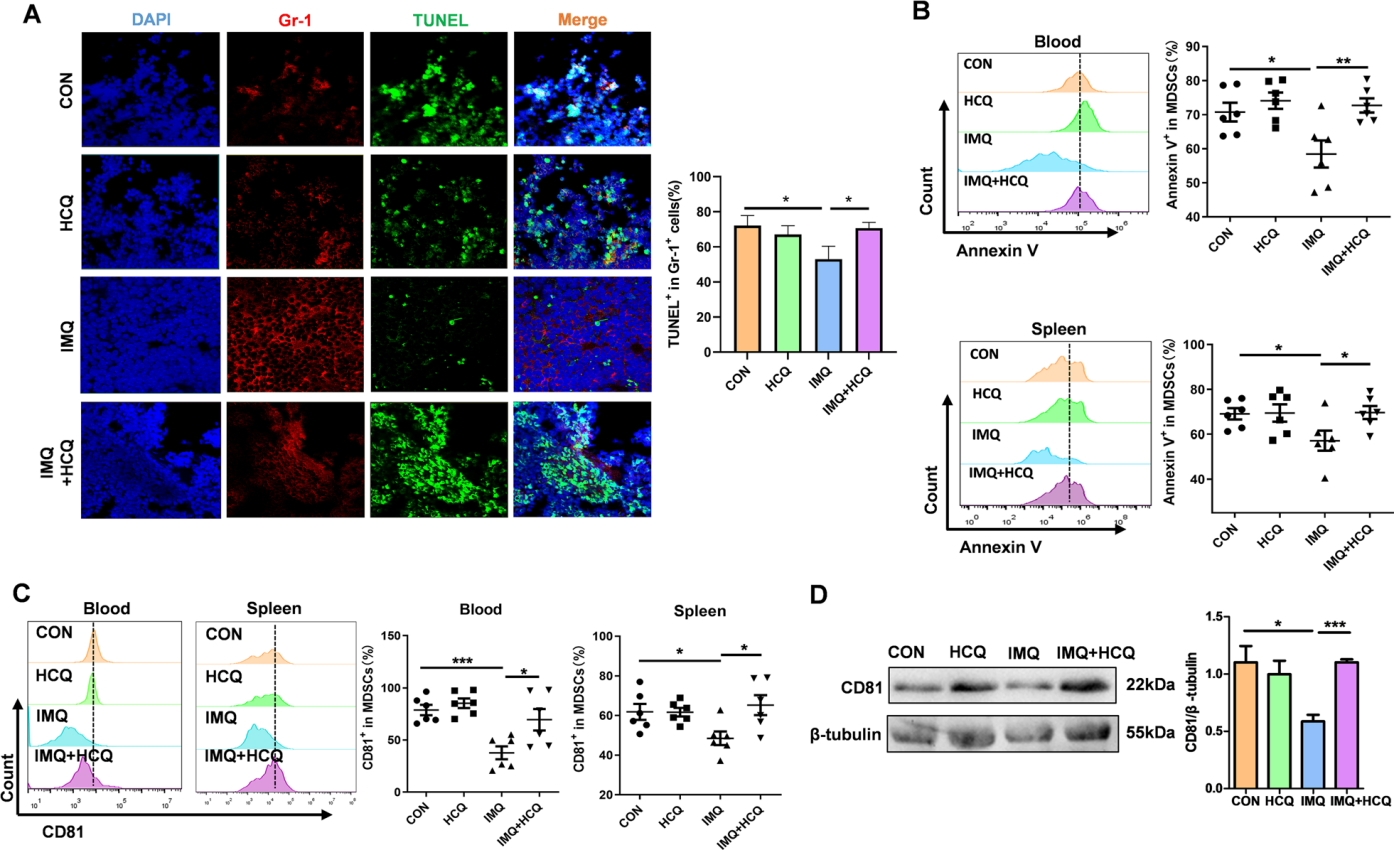
HCQ induces apoptosis of MDSCs and increases the level of CD81 in MDSCs. **A** Immunofluorescence detection of the apoptotic effects of HCQ on Gr-1^+^ cells in spleen. Scale bar = 50 μm (n = 3 mice/group). **B** The percentage of Annexin V^+^ in MDSCs in blood and spleen was determined by FACS (n = 6 mice/group). **C** The percentages of CD81^+^ in MDSCs were determined by FACS in blood and spleen (n = 6 mice/group). **D** Western blot was used to detect the protein expression of CD81 in purified MDSCs isolated from spleen (n = 4 mice/group). Data are presented as the mean ± standard error of the mean (SEM). *P ≤ 0.05, **P ≤ 0.01, ***P ≤ 0.001

Apoptosis regulates the persistence of MDSCs during inflammation, which is related to viability and proportion of MDSCs (Li et al. 2018; Zhang et al. 2018a, b; Yang et al. 2019). To investigate the mechanism of HCQ affecting MDSCs, we found that apoptosis of MDSCs increased by HCQ adequately. First, immunofluorescence assay was used to label Gr-1-positive cells in the cryopreserved spleen tissues. Alexa Fluor 488-conjugated-TUNEL dye indicated the apoptotic Gr-1-positive cells. The results confirmed that mice in the HCQ-treated lupus group were predisposed with the induction of apoptosis in Gr-1-positive cells compared with that in the IMQ-only group (Fig. 2A). We next detected the proportion of Annexin V^+^ MDSCs in the blood and spleen of mice. As the results shown, the proportion of Annexin V^+^ MDSCs decreased in lupus mice compared with that in healthy control (*P* < 0.05), whereas HCQ treatment rescued the downside apoptosis (*P* < 0.01) (Fig. 2B). MDSCs were further isolated from spleen (Fig. 1C). We also examined the expression levels of apoptosis-related markers in purified MDSCs by Western blotting, including caspase-3, cleaved caspase-3, B-cell lymphoma-extra-large (Bcl-XL) and Bcl-2-associated X protein (Bax). The expression level of caspase-3 and cleaved caspase-3 in MDSCs increased in HCQ-treated mice (*P* < 0.05). Bcl-XL, one of the members of anti-apoptotic family, was elevated in MDSCs of lupus mice and decreased after HCQ treatment. The expression level of Bax was higher in the HCQ-treated lupus group (*P* < 0.05) (Additional file 1: Fig. S3A). All features indicated that HCQ could increase the apoptosis of MDSCs in mice with lupus.

To identify the target regulating the apoptosis of MDSCs, we detected the expression level of apoptosis-related genes in MDSCs. The results showed that the expression level of CD81 was significantly reduced in lupus mice, which suggested that CD81 might play a crucial role in the apoptosis of MDSC (Additional file 1: Fig. S3B). In our research, we observed that HCQ treatment significantly increased the expression level of CD81 in MDSCs, no matter in blood or in spleen (Fig. 2C). Besides, the expression level of CD81 in purified MDSCs from murine spleens was also up-regulated in the HCQ-treated lupus group, compared with that in lupus group (*P* < 0.05) (Fig. 2D).

**Fig. 3:**
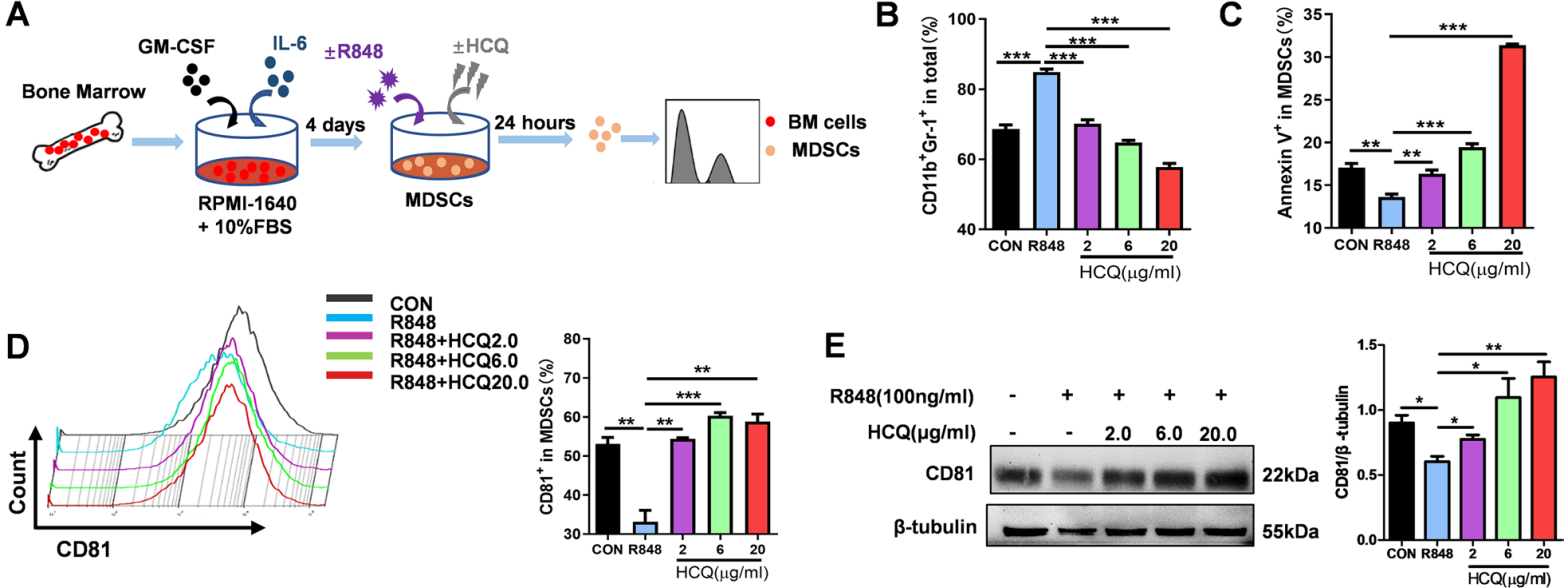
HCQ induces the apoptosis of BM-derived MDSCs under stimulation with R848 and reverses the expression of CD81 in MDSCs. **A** The scheme showing the experimental design. Cells from bone marrow (BM) were stimulated by GM-CSF (40 ng/mL) and IL-6 (40 ng/mL) for 4 days, then, treated with or without R848 (100 ng/mL) and HCQ (2, 6, 20 µg/mL), and after 24 h, the cells were harvested for detection. **B**, **C**. The proportions (**B**) and apoptotic rate (**C**) of bone marrow-derived CD11b^+^Gr-1^+^ MDSCs were determined by FACS (n = 4). **D** The percentages of CD81^+^ in MDSCs were determined by FACS in BM-derived MDSCs (**C**) (n = 4). **E** Western blot was used to detect the expression of CD81 protein in BM-derived MDSCs (n = 4). Data are presented as the mean ± standard error of the mean (SEM). *P ≤ 0.05, **P ≤ 0.01, ***P ≤ 0.001

Furthermore, as illustrated in Fig. 3A, BM cells were acquired from normal mice and cultured with GM-CSF and IL-6 to induce MDSCs, namely BM-derived MDSCs (BM-MDSCs), in vitro*.* To generate lupus environment, BM-MDSCs were stimulated with treated with a TLR7 agonist R848 (100 ng/mL), accompanied by different concentrations of HCQ (2, 6, and 20 µg/mL). The number of MDSCs was decreases by HCQ in a dose-dependent manner (Fig. 3B). As expected, HCQ showed an obvious promoting effect on the percentage of Annexin V^+^ BM-MDSCs, and the most significant effect appeared at the concentration of 20 µg/mL (*P* < 0.001) (Fig. 3C). The expression levels of apoptosis-related markers in BM-MDSCs were determined as well. The expression level of Bcl-XL in BM-MDSCs was highly inhibited by HCQ, whereas the expression levels of Bax, caspase-3, and cleaved caspase-3 were significantly induced by HCQ treatment (Additional file 1: Fig. S4). We examined genes associated with apoptosis and also found significant differential expression of CD81 after R848 administration (Additional file 1: Fig. S3C). The percentage of CD81^+^ MDSCs was increased by HCQ under R848 treatment, detected by flow cytometry (Fig. 3D). What’s more, a dose-dependent promotion on CD81 protein was found under various concentrations of HCQ treatment by western blot (Fig. 3E). These findings were consistent with those achieved in vivo, and further demonstrated that HCQ could promote the apoptosis of MDSCs in SLE. Taken together, we supposed that the promotion of HCQ on the apoptosis of MDSCs might be related to CD81.

### CD81 plays a crucial role in HCQ treatment by increasing the apoptosis of MDSCs

**Fig. 4:**
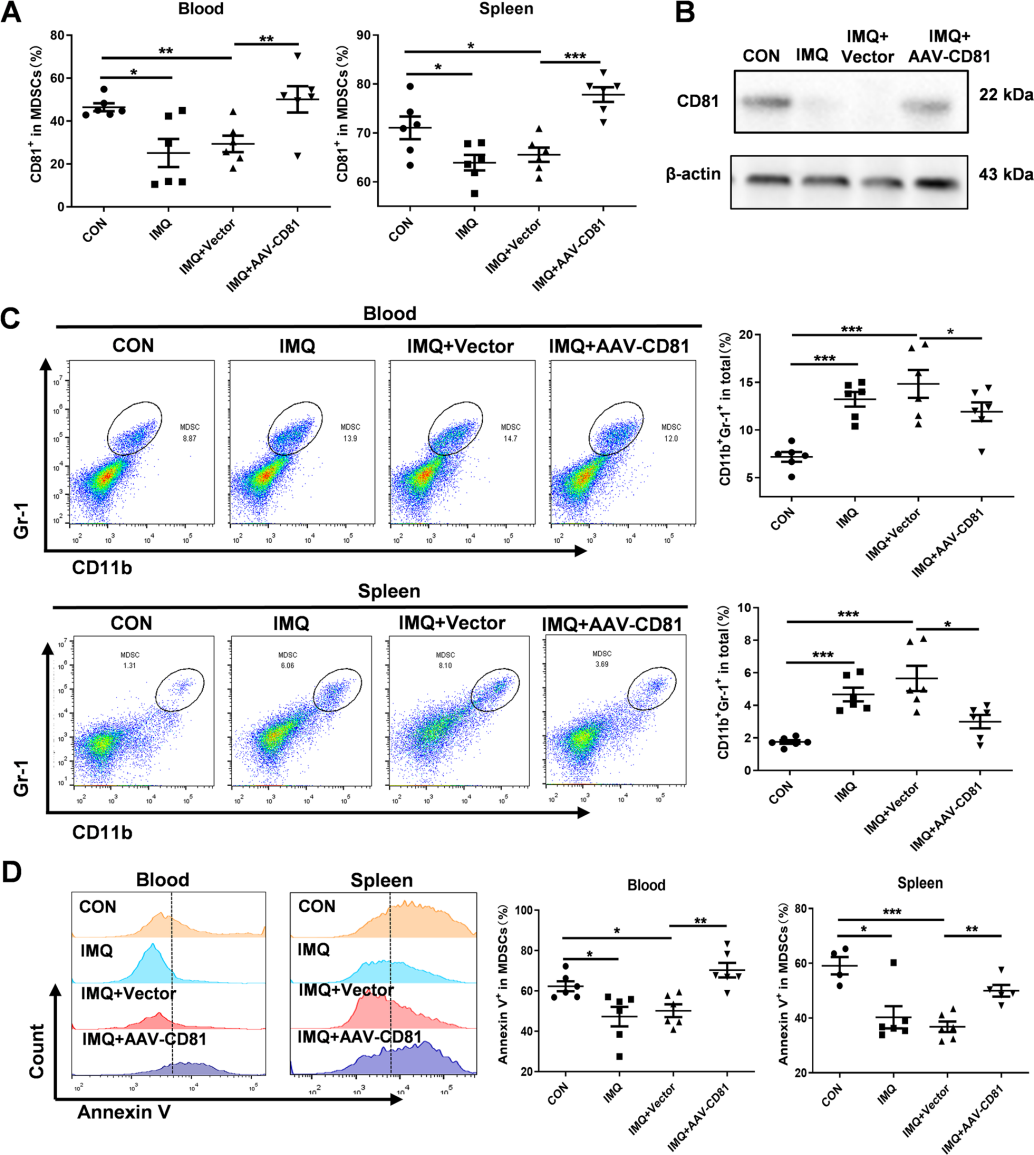
CD81 plays a crucial role in HCQ treatment by increasing the apoptosis of MDSCs. **A**, **B** The expression level of CD81 in MDSCs of blood and spleen was determined by FACS (A) and Western blotting (**B**) (n = 6 mice/group). **C** The number of MDSCs in blood and spleen was measured by FACs and compared with that of achieved statistically (n = 6 mice/group). **D** The percentage of Annexin V^+^ in MDSCs in blood and spleen was determined by FACs (n = 6 mice/group). Data are presented as the mean ± standard error of the mean (SEM). *P ≤ 0.05, **P ≤ 0.01, ***P ≤ 0.001

We then investigated the concentrate role of CD81 in MDSCs in vivo. CD81-overexpressed adeno-associated virus was intraperitoneally injected at the beginning of establishing the IMQ-induce lupus model. In the IMQ + AAV-CD81 group, the proportion of CD81^+^ in MDSCs was highly elevated compared with that in the IMQ + Vector group in both blood and spleen (Fig. 4A), and we found the similar trend in MDSCs isolated from spleen (Fig. 4B). These results confirmed that the mouse model of CD81 overexpression was successfully constructed. To explore the effects of CD81 on MDSCs, we first assessed its suppressive effects on accumulation of MDSCs. The total number of CD11b^+^Gr-1^+^ MDSCs increased in the IMQ + Vector group, while overexpression of CD81 significantly decreased the number of MDSCs in blood and spleen (Fig. 4C).

Furthermore, as data displayed in Fig. 4D, CD81 overexpression remarkably increased the percentage of Annexin V^+^ in MDSCs in blood and spleen, compared with that in the IMQ + Vector group. The expression levels of apoptosis-related markers also changed as expected. When CD81 was overexpressed, the expression level of Bcl-xl in isolated MDSCs was decreased, whereas the expression levels of Bax, Caspase3 and Cleaved caspase3 were significantly induced (Additional file 1: Fig. S4A).

### Lupus-like syndrome is attenuated by overexpression of CD81 in mics with lupus

**Fig. 5:**
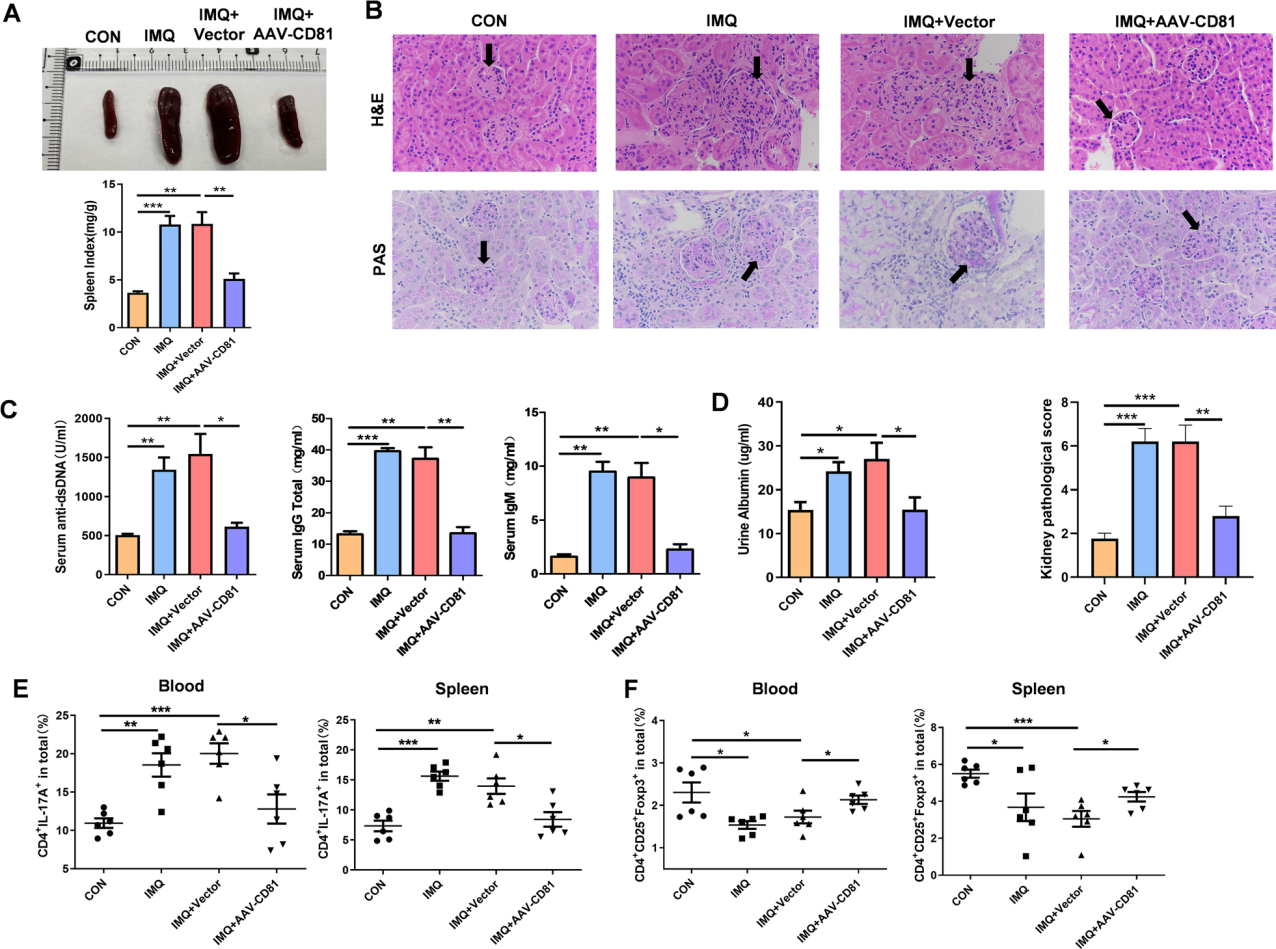
Lupus-like syndrome is attenuated in lupus mice by overexpression of CD81. **A** Representative photographs of the spleen from control groups, IMQ groups, IMQ + vector groups and IMQ + AAV-CD81 groups (n = 4 mice/group). **B** H&E and PAS staining of kidney sections in each group (scale bar = 50 μm). (n = 4 mice/group). **C**, **D** The levels of serum anti-dsDNA, IgG and IgM (**C**) and urine protein (**D**) were detected by ELISA from control groups, IMQ groups, IMQ + vector groups and IMQ + AAV-CD81 groups (n = 4 mice/group). **E**, **F** The proportions of Th17 and Treg cells in blood (**E**) and spleen (**F**) were determined by FACS (n = 6 mice/group). Data are presented the mean ± standard error of the mean (SEM). *P ≤ 0.05, **P ≤ 0.01, ***P ≤ 0.001

We surprisingly found that overexpression of CD81 ameliorated the lupus-like syndrome directly. As results shown, CD81 improved splenomegaly (Fig. 5A). HE and PAS staining of kidney sections also showed that CD81 mitigated the glomerulonephritis, mesangial matrix diffuse expansion and infiltration of lymphocytes (Fig. 5B). The serum levels of IgG, IgM, anti-dsDNA and urine protein were lower in the IMQ + AAV-CD81 group, compared with those in the IMQ + Vector group (Fig. 5C, D). In addition, we observed the effects of CD81 on Th17 and Treg cells, which CD81 overexpression recovered the increased proportion of Th17 cells and decreased proportion of Treg cells in blood and spleen of mice with lupus (Fig. 5E, F). In summary, CD81 could relieve the imbalance of Th17/Treg cells in IMQ-induced lupus mice, which might contribute to the regulation of the functions of MDSCs.

### HCQ rescues the expression level of CD81 to increase MDSC apoptosis

**Fig. 6:**
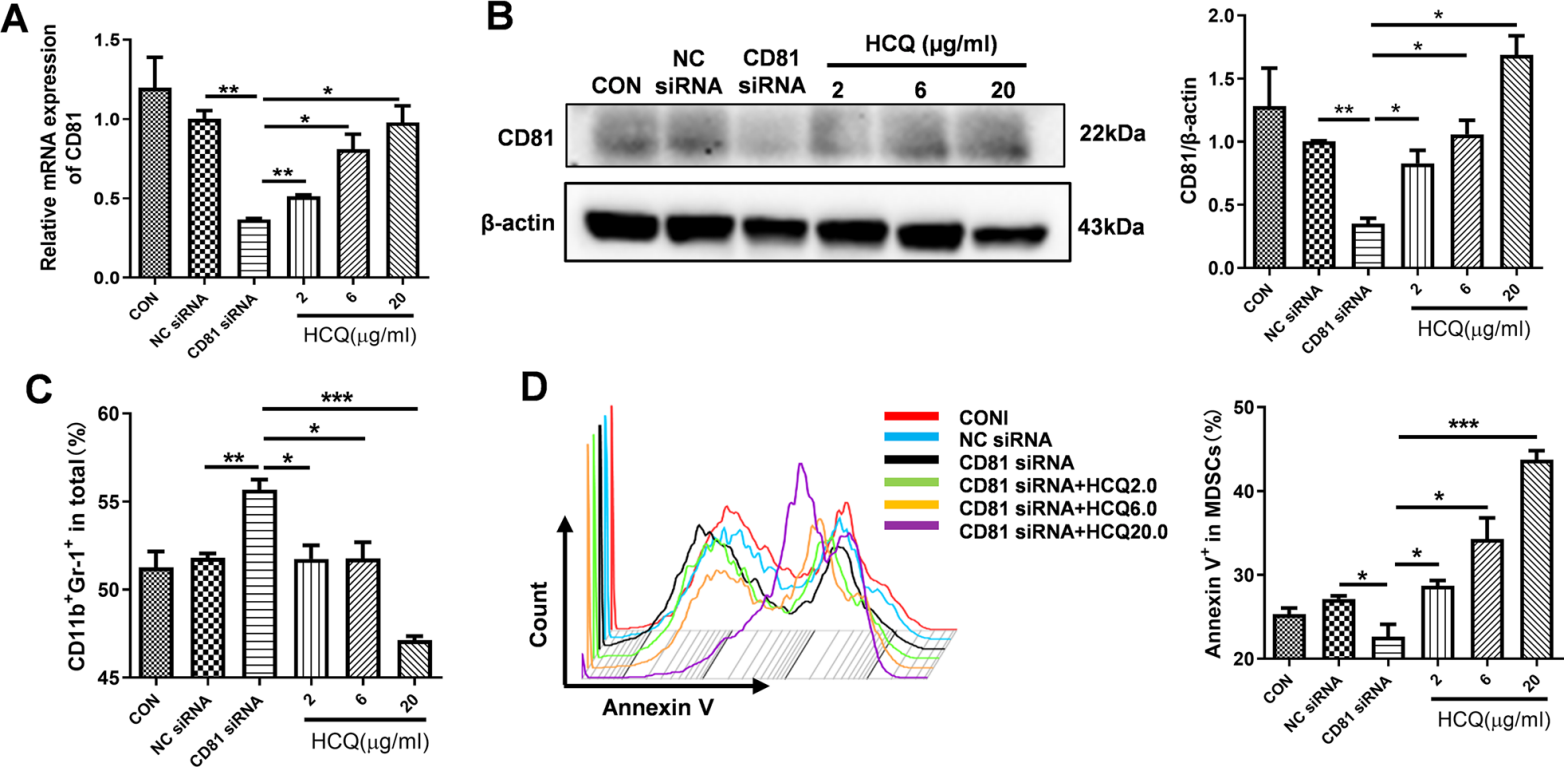
HCQ rescues the expression levels of CD81, leading to increase the apoptosis of MDSCs. **A**, **B** qRT-PCR (**A**) and Western blotting (**B**) was used to detect the expression level of CD81 in BM-derived MDSCs (n = 4). **C** The number of BM-derived MDSCs was determined by FACS (n = 4). **D** The effects of HCQ on the apoptosis of BM-MDSCs after interfering CD81 were measured by FACS (n = 4). Data are presented as the mean ± standard error of the mean (SEM). *P ≤ 0.05, **P ≤ 0.01, ***P ≤ 0.001

To further verify the relationship between HCQ and CD81 in the apoptosis of MDSCs, we used small interfering RNA (siRNA) to knock down the expression level of CD81 in BM-MDSCs and evaluated the effects of HCQ on the expression level of CD81. We chose one of the three siRNAs according to the silencing efficiency. After simultaneous treatment, the results showed that HCQ concentration was directly proportional to the increased expression level of CD81 in MDSCs (Fig. 6A). CD81 protein was inhibited by CD81 siRNA and HCQ could strongly reverse it (Fig. 6B). These findings suggested that the induction in the apoptosis of MDSCs might be achieved by promoting the expression level of CD81 in HCQ-treated group.

Since HCQ could up-regulate the expression level of CD81, we further investigated the effects of HCQ on the apoptosis of MDSCs and immune balance of Th17/Treg cells when CD81 was interfered. The massive accumulation of BM-MDSCs was caused by CD81 siRNA, and reduced by HCQ treatment (Fig. 6C). As expected, HCQ treatment could significantly increase the percentages of Annexin V in BM-MDSCs when transfecting BM-MDSCs with CD81 siRNA (Fig. 6D) and altered the expression levels of apoptosis-related proteins in BM-MDSCs (Additional file 1: Fig. S4B). Subsequently, we co-cultured normal mouse splenocytes with BM-MDSCs treated for 72 h. The results revealed that CD81-interfered BM-MDSCs impaired balance of Treg/Th17 cells, while the imbalance was reversed in HCQ-treated BM-MDSCs compared with that in CD81-interfered BM-MDSCs group. HCQ increased the proportion of Treg cells and decreased the differentiation of Th17 cells (Additional file 1: Fig. S5A, B). These findings supported that HCQ induced the apoptosis of MDSCs and relieved immune imbalance of Th17/Treg cells through up-regulating the expression level of CD81.

### Molecular docking of HCQ with CD81

**Fig. 7:**
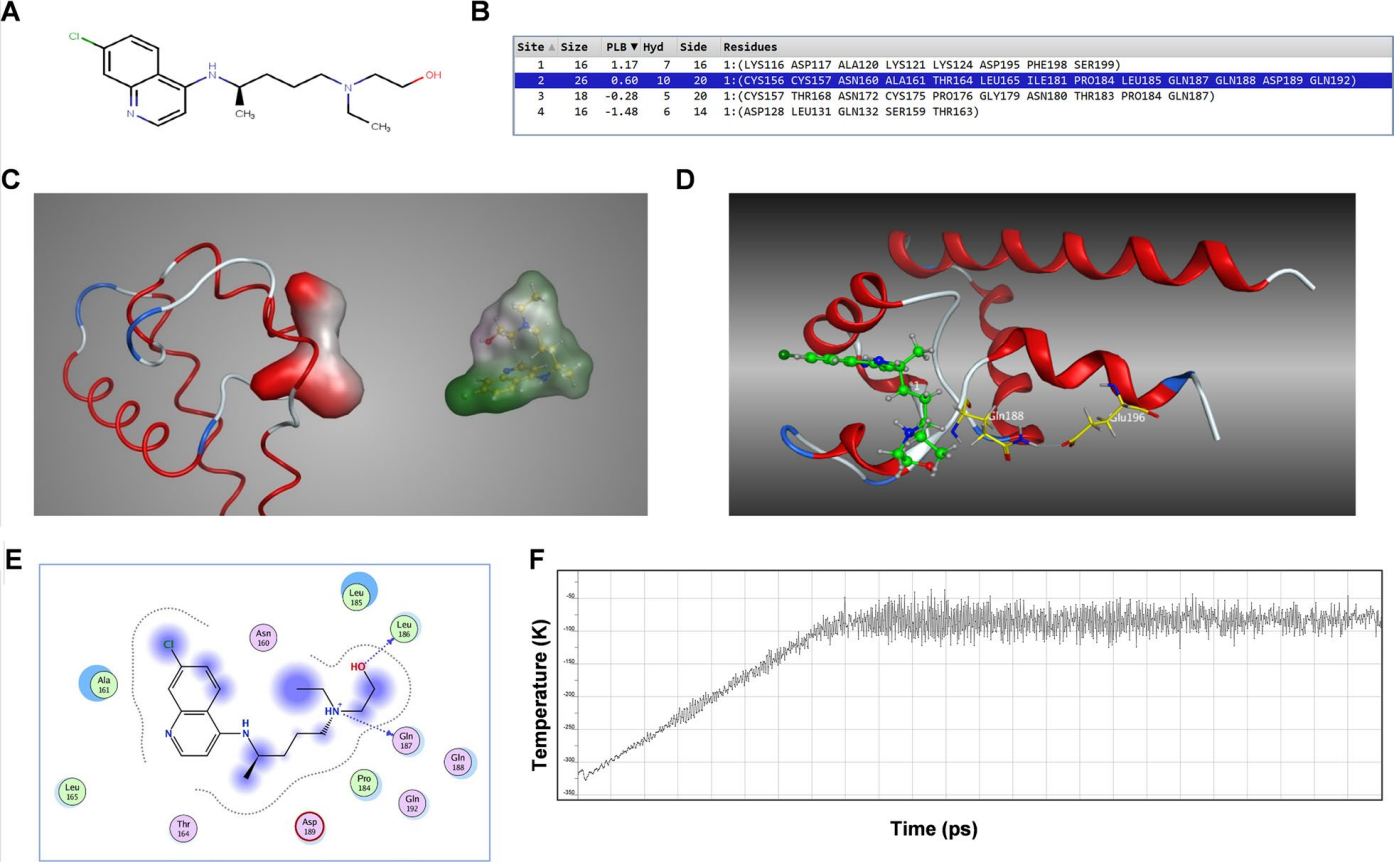
Molecular docking of HCQ with CD81. **A** The 2D structure of HCQ. **B** Four candidate binding sites in CD81. **C** A comparison of shape between 2nd binding site and HCQ. **D** 3D docking results of HCQ with CD81. **E** The 2D binding mode of HCQ with CD81. **F** Potential energy curve of a 0.6 nm MD of the complex of HCQ with CD81

In order to explore the relationship between HCQ and CD81 in depth, we performed molecular docking. The two-dimensional (2D) structure of HCQ is shown in Fig. 7A. MOE's Site Finder falls into the category of geometric methods as no energy models were used. Alternatively, the relative positions and accessibility of the receptor atoms were considered along with a rough classification of chemical type. The Site Finder methodology was based on Alpha Shapes which were a generalization of convex hulls developed by Edelsbrunner in 1995.

In total, 4 possible sites were found by MOE. The detailed data of the sites were depicted in Fig. 7B. Rank sites according to their Propensity for Ligand Binding (PLB) score. The size and shape of the 2nd site were close to ligand HCQ (Fig. 7C). Thus, this binding site was selected for the following docking experiment.

To investigate the binding mode of HCQ with CD81, docking experiments were carried out. The docking score of HCQ was − 70.01 kcal/mol for CD81. This computational result indicated that HCQ could well interact with CD81. The binding mode of HCQ with CD81 was illustrated in Fig. 7D, E. To confirm the results of the docking experiments, a short molecular dynamic (MD) simulation was carried out. The MD includes 3 stages, the first stage is heating the system from 0 to 300 K, followed by equilibration, and finally a production stage. The curve of atomic potential energy (Fig. 7F) indicated that the results of docking experiments were relatively steady. Such molecular docking results confirmed that HCQ well interacted with CD81 and the combinatory effect was relatively steady.

## Discussion

Recent studies have described the pharmacokinetics, pharmacodynamic properties, clinical efficacy and safety of HCQ in the treatment of SLE (Carter et al. 2016; Olsen et al. 2013; Fanouriakis et al. 2019). The underlying molecular mechanisms that mediate the effect of HCQ on SLE have been poorly understood. In the pathological process of SLE, our sight is focused on MDSCs, which accumulated in SLE patients and lupus mice (Li et al. 2019a, b; Ji et al. 2016; Li et al. 2019a, b). MDSCs are perceived as a myeloid-derived heterogeneous cell population composed of immature macrophages, granulocytes and dendritic cells. Immune suppression is the major function of MDSCs in the pathogenesis of cancer, infectious diseases and autoimmune disorders, including inhibiting the proliferation of T cells, accompanied with the upregulation of arginase, NO, ROS and prostaglandin E2 (PGE2) (Groth et al. 2019). It’s clearly that accumulation of MDSCs inhibit tumor immune responses in antigen-specific and non-specific manners, and induce tumor angiogenesis capability, which promote tumor progression (Umansky et al. 2016). Unlike the deleterious role of MDSCs in tumor, it would be an enormous challenge to confirm a unifying hypothesis showing the functions of MDSCs in various autoimmune diseases. MDSCs were involved in the disease course of SLE, also accompanied with the arguable function (Wang et al. 2021; Zhang et al. 2014). Iwata et al. have identified the accumulation of MDSCs in the MRL-Fas lpr mice for the first time (Iwata et al. 2010). Some studies indicated that MDSCs induced expansion of regulatory B cells via iNOS, and inhibited the cytokine-mediated differentiation of naïve B cells into plasma cells, to relieved the injuries in lupus mice (Trigunaite et al. 2013). What’s more, infusion of MDSCs derived from C57BL/6 mice reduced the levels of anti-dsDNA antibody in serum and proteinuria in roquin^san/san^ lupus mice (Park et al. 2016). While other studies pointed out the opposite function of MDSCs in lupus. Wu et al. have reported that the increased MDSCs in peripheral blood of SLE patients were positively correlated with the severity of lupus (Wu et al. 2016). We have also found that MDSCs promoted Th17 polarization and inhibited Treg differentiation by IL-1β secretion to aggravate disease in MRL/lpr lupus mice (Ji et al. 2016). Overall, the role of MDSCs in the disease mechanism of SLE remains controversial and intangible.

In our study, we found that HCQ inhibited the expansion of MDSCs in IMQ-induced lupus mice. The same results were obtained in vitro, which stimulated BM-MDSCs with R848 to generate lupus environment. R848 could increase the number of MDSCs, and different concentrations of HCQ decreased it without a dose-dependent effect. These phenomena hint the crucial function of MDSCs involved in the course of SLE. To figure out the reason for the changes in the number of MDSCs, we detected relevant signaling molecules of MDSCs, and found that the apoptosis of MDSCs was significantly influenced after HCQ treatment. Combined with the expression levels of apoptosis-related genes, HCQ was found to have a facilitation effect on the apoptosis of MDSCs in vivo and in vitro. The results were consistent with the decreased number of MDSCs after HCQ treatment.

Furthermore, we found that CD81 plays an important role in the regulation of MDSC apoptosis. Some studies demonstrated that lack of CD81 expression was related to SLE disease activity, and it might be a potential marker for the diagnosis of SLE patients (Abu-Zahab et al. 2017). HCQ recovered the expression level of CD81 in MDSCs of mice with lupus. CD81 overexpression increased the apoptosis of MDSCs, and interfering the expression level of CD81 in MDSCs showed opposite results. Besides, CD81 overexpression alleviated the disease activity of lupus mice directly, as illustrated by improved indexes of pathology. All outcomes verified the key role of CD81 in the apoptosis of MDSCs. It is noteworthy that HCQ could rescue the low-level of the expression level of CD81 induced by CD81 siRNA in MDSCs. Taken together, the increase of apoptosis of MDSCs is directly correlated to CD81 signaling pathway in the treatment of lupus with HCQ. What’s more, in terms of molecular structure, we found that the direct binding mode and affinity between HCQ and CD81, which confirmed that HCQ could target CD81 and interact well with CD81, which indicated that HCQ might induced the expression of CD81 in two ways—a direct bonding manner and an indirect manner through signaling pathways.

In our study, HCQ not only affected the apoptosis of MDSCs, but also influenced their immunosuppressive functions in mice with lupus. HCQ weakened the production of ROS, and affected the proliferation rate of CD4^+^ T cells. The capacity of MDSCs to regulate the balance of Th17/Treg cells might be a critical pathogenic factor in SLE, thus, we counted the number of Th17 and Treg cells in lupus mice, and confirmed that HCQ could improve the immune imbalance of Th17/Treg cells through increasing the proportion of Treg cells and decreasing the differentiation of Th17 cells. A previous study also confirmed that HCQ inhibit differentiation of Th17 cell and production of IL-17 (Yang et al. 2018). Meanwhile, when CD81 was over-expressed in lupus mice, the number of Th17 cells was reduced, and Treg cells expanded. The alteration in immune balance corresponded to relief of SLE symptoms. In general, HCQ relieved immune imbalance of Th17/Treg cells by up-regulating the expression level of CD81. These findings indicated that various functions of MDSCs are involved in the course of SLE. MDSCs proved to be extremely important in autoimmune disease.

Taking all these factors into consideration, MDSCs serve as captivating targets for immunotherapy by suppressing T cell activation and function, which play two opposite effects in the regulation of immune balance. We have demonstrated that HCQ mitigated SLE by target a series of anti-inflammatory pathways involving MDSC and its immunosuppressive function. Our research confirmed the crucial participation of MDSCs in SLE, which provided novel therapeutic modality to treat SLE and other autoimmune diseases.

## Conclusion

In summary, our study revealed that the mechanism of alleviation of HCQ on SLE might be attributed to the induction of apoptosis of MDSCs through regulating the expression level of CD81 in MDSCs. The results may present new insights into the immunomodulatory potency of hydroxychloroquine and provide the rationale to search for more potent and/or selective inhibitors for therapeutic targets. Further research is essential to investigate the immunosuppressive functions of MDSCs.

## Supplementary Information

**Additional file 1: Figure S1.** Effects of HCQ on IMQ-induced mice. **Figure S2.** HCQ regulated the immunosuppressive function of BM-MDSCs in vitro. **Figure S3.** The effect of HCQ on apoptosis-related genes of MDSCs in lupus environment. **Figure S4.** The effect of CD81 on apoptosis-related genes of MDSCs in lupus environment. **Figure S5.** CD81 upset the balance of Th17/Treg cells, and HCQ recovered it. **Table S1.** Primer sequences used for the qRT-PCR analysis.

## Data Availability

The data used to support the findings of the present study are available from the corresponding author upon request.

## Abbreviations

SLE: Systemic lupus erythematosus
HCQ: Hydroxychloroquine
MDSC: Myeloid-derived suppressor cell
IL: Interleukin
IFN: Interferon
ROS: Reactive oxygen species
Th: T helper cell
Treg: Regulatory T cell
IMQ: Imiquimod
iNOS: Nitric oxide synthase
TGF-β: Transforming growth factor-β
BM: Bone marrow
CFSE: Carboxyfluorescein succinimidyl ester
LPS: Lipopolysaccharide
siRNA: Small interfering RNA
TUNEL: TdT mediated dUTP Nick End Labeling
PMA: Phorbol-12-myristate-13-acetate
Ion: Ionomycin
BFA: Brefeldin A
