# Supplementary material for: Hydroxychloroquine induces apoptosis of myeloid-derived suppressor cells via up-regulation of CD81 contributing to alleviate lupus symptoms
Source: Mol Med. 2022 Jun 15;28:65. doi: 10.1186/s10020-022-00493-6 (PMC9199128; doi:10.1186/s10020-022-00493-6)

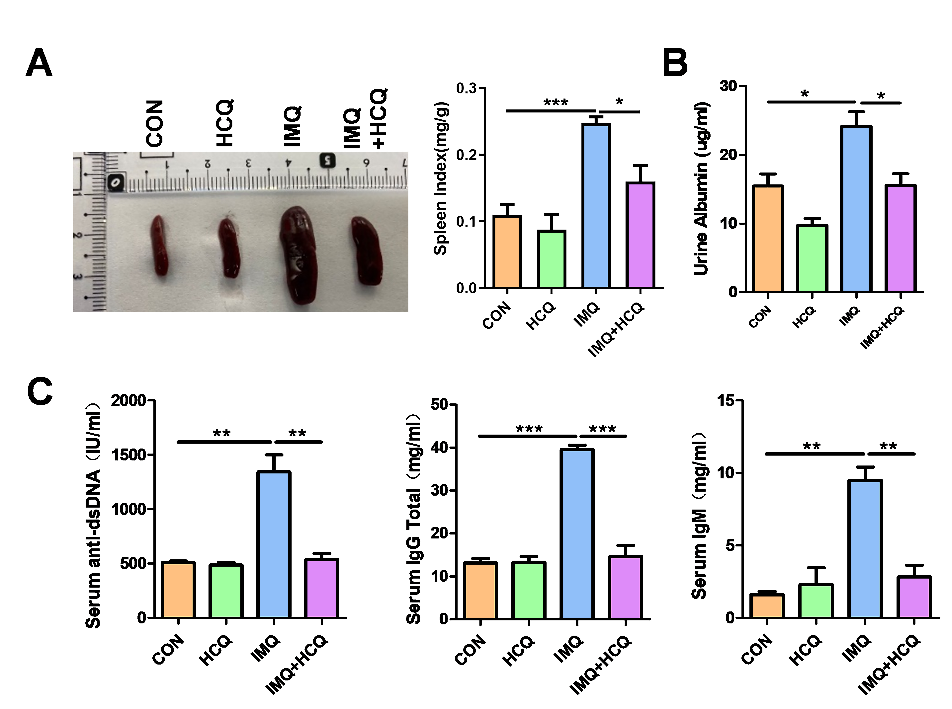


**Figure S1. Effects of HCQ on IMQ-induced mice.** (A) Representative photographs of the spleen and the spleen weight from control groups, IMQ groups, HCQ groups and IMQ+HCQ groups (n=4 mice/group), representative for four mice analyzed. (B, C) The levels of urine protein (B), serum anti-dsDNA, IgG and IgM (C) were detected by ELISA from control groups, IMQ groups, HCQ groups and IMQ+HCQ groups (n=4 mice/group). Data represent the mean scores ± SEM. *P ≤ 0.05, **P ≤ 0.01, ***P ≤ 0.001.


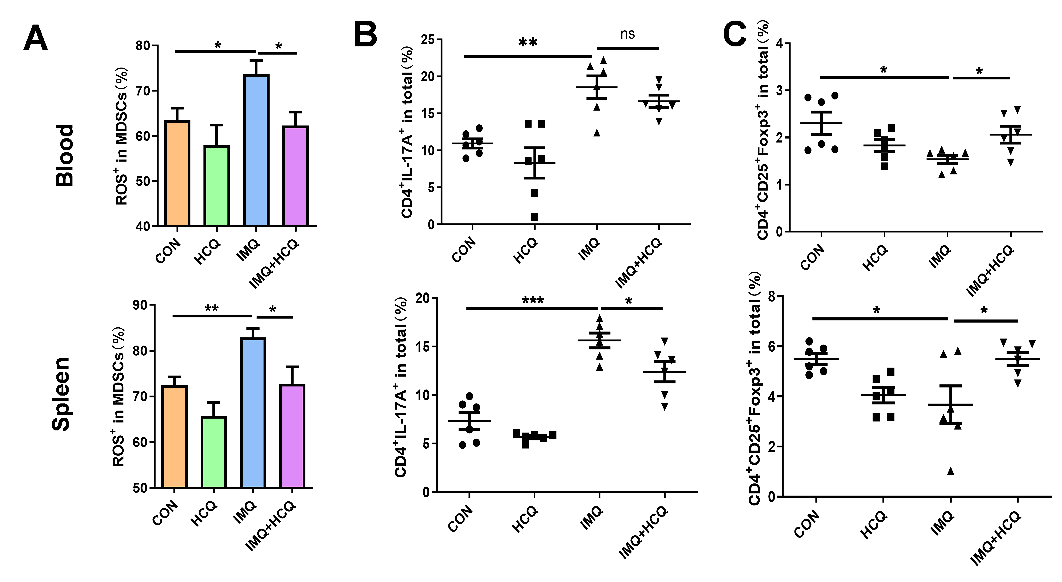


**Figure S2. HCQ regulated the immunosuppressive function of BM-MDSCs in vitro.** (A) The production levels of ROS in MDSCs in blood and spleen were determined by FACS. (B, C). The frequency of Th17 cells (B) and Treg cells (C) in blood and spleen from control groups, IMQ groups, HCQ groups and IMQ+HCQ groups (n=6 mice/group). Data represent the mean scores ± SEM. *P ≤ 0.05, **P ≤ 0.01, ***P ≤ 0.001.


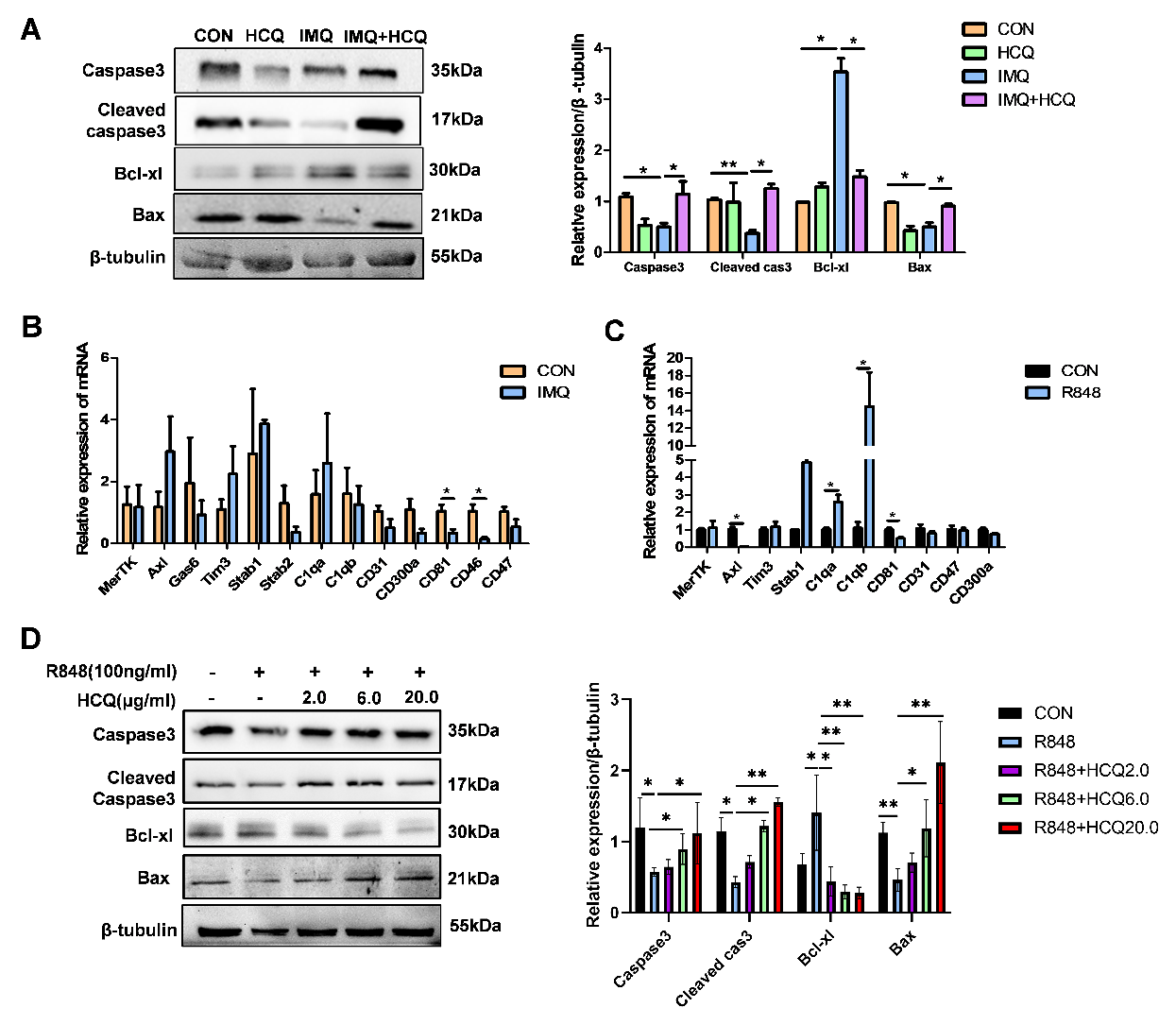


**Figure S3.** **The effect of HCQ on apoptosis-related genes of MDSCs in lupus environment.** (A) The protein expression levels of apoptosis-related markers, including caspase-3, cleaved caspase-3, Bcl-xl, and Bax in MDSCs isolated from spleen were detected by Western blotting (n=3 mice/group). **(**B) The mRNA expression of apoptosis-related genes in MDSCs isolated from spleen of control and IMQ-induced mice (n=3 mice/group). (C)The mRNA expression of apoptosis-related genes in BM-MDSCs treated with or without R848 (n=3). (D)The protein expression levels of apoptosis-related markers, including caspase-3, cleaved caspase-3, Bcl-xl, and Bax in BM-MDSCs treated with or without R848 and different concentrations of HCQ (n=3). Data represent the mean scores ± SEM. *P ≤ 0.05, **P ≤ 0.01.


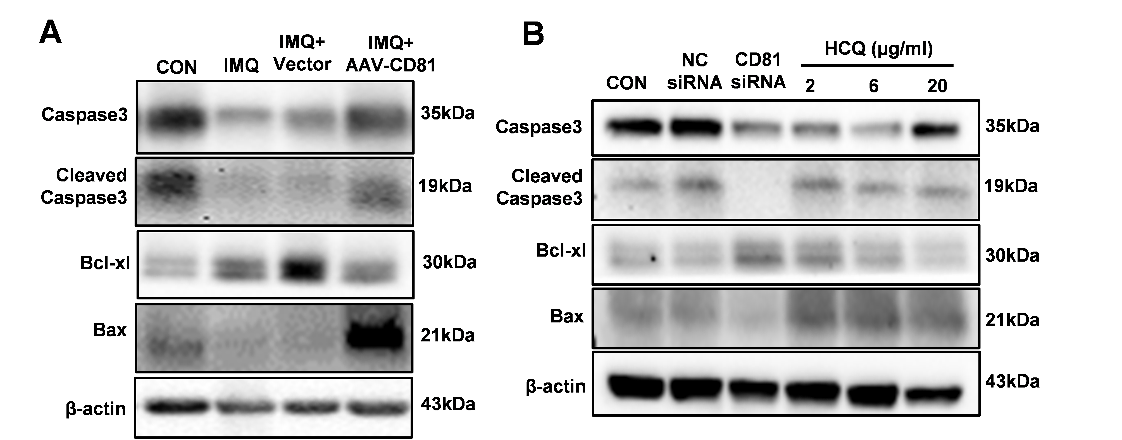


**Figure S4. The effect of CD81 on apoptosis-related genes of MDSCs in lupus environment.** (A) The protein expression levels of apoptosis-related markers, including caspase-3, cleaved caspase-3, Bcl-xl, and Bax in MDSCs isolated from spleen were detected by Western blotting (n=4 mice/group). **(**B) The protein expression levels of apoptosis-related markers, including caspase-3, cleaved caspase-3, Bcl-xl, and Bax in BM-MDSCs transfected with CD81siRNA(n=3).


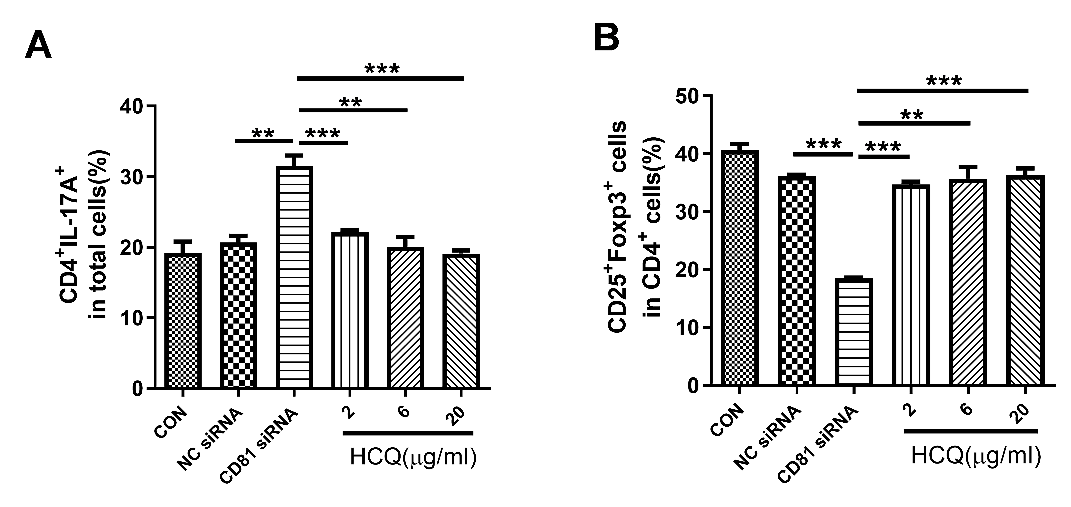


**Figure S5. CD81 upset the balance of Th17/Treg cells, and HCQ recovered it.** (A, B) The effects of HCQ on the differentiation of MDSCs-Th17 (A) and Treg (B) cells after interfering CD81 siRNAwas measured by FACS (n=3). Data are presented as the mean ± standard error of the mean (SEM). *P ≤ 0.05, **P ≤ 0.01, ***P ≤ 0.001.

**Table S1. Primer sequences used for the qRT-PCR analysis**


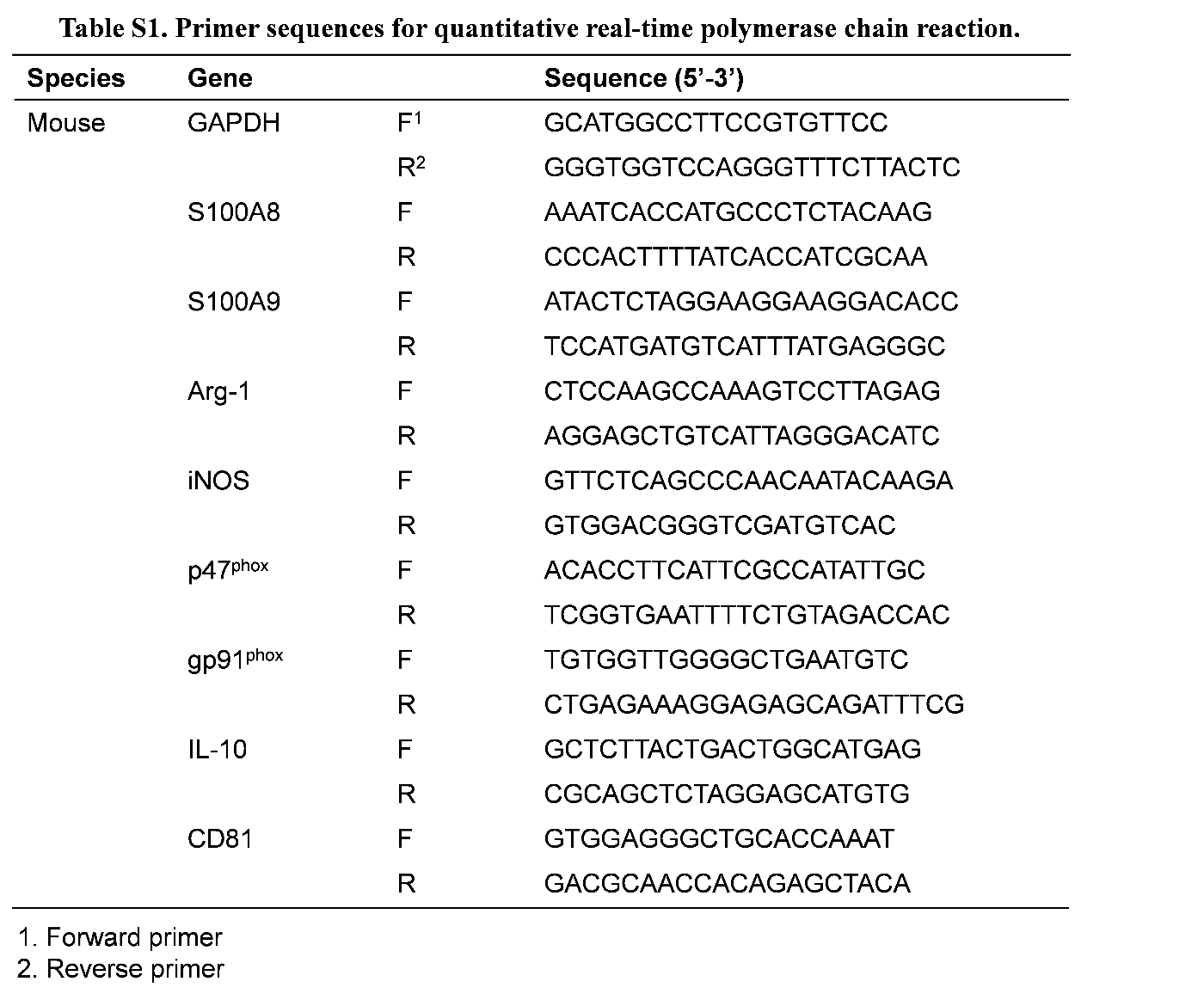

Supplement: Supplementary file 1 — Additional file 1: Figure S1. Effects of HCQ on IMQ-induced mice. Figure S2. HCQ regulated the immunosuppressive function of BM-MDSCs in vitro. Figure S3. The effect of HCQ on apoptosis-related genes of MDSCs in lupus environment. Figure S4. The effect of CD81 on apoptosis-related genes of MDSCs in lupus environment. Figure S5. CD81 upset the balance of Th17/Treg cells, and HCQ recovered it. Table S1. Primer sequences used for the qRT-PCR analysis. [file 10020_2022_493_MOESM1_ESM.docx]
